# Predictive factors of ovarian carcinoma for women with ovarian endometrioma aged 45 years and older in China

**DOI:** 10.1186/s13048-017-0343-2

**Published:** 2017-07-17

**Authors:** Zheng-Xing He, Hong-Hui Shi, Qing-Bo Fan, Lan Zhu, Jin-Hua Leng, Da-Wei Sun, Zhan-Fei Li, Keng Shen, Shu Wang, Jing-He Lang

**Affiliations:** Department of Gynecology and Obstetrics, Peking Union Medical College Hospital (PUMCH), Chinese Academy of Medical Sciences (CAMS) & Peking Union Medical College, 1 ShuaiFuYuan, DongCheng District, Beijing, 100730 People’s Republic of China

**Keywords:** Endometriosis, Ovarian carcinoma, Risk factor

## Abstract

**Background:**

To explore the risk factors of endometriosis-associated ovarian cancer (EAOC) in women with ovarian endometriosis (OEM) aged 45 years and above in China.

**Methods:**

This study reviewed the medical records of 1038 women in total who were aged 45 years and above, surgical-pathologically diagnosed with ovarian endometriosis, and were treated at Peking Union Medical College Hospital between December 1996 and December 2016. Histology evaluation was used to determine whether the ovarian endometriosis specimen was with (*n* = 30) or without (*n* = 1008) ovarian cancer.

**Results:**

2.9% (30/1038) of women with the surgical-pathological diagnosis of ovarian endometriosis were found to have EAOC. Those patients with EAOC were prone to be in the postmenopausal status at the time of the diagnosis (OR 5.50, 95%CI 2.54–11.90, *P* < .001) and larger size of tumor (≥8 cm, OR 7.19, 95% CI 3.34–15.50, *P* < .001), and higher prevalence of coexisting with endometrial disorders (OR 4.11, 95%CI 1.73–9.73, *P* = .003). This study showed that patients of an older age when diagnosed with OEM, were at a higher risk of developing EAOC, respectively measuring of 1.7% (13/751) at 45–49 years, 5.6% (12/215) at 50–54 years, and 10.0%(5/50) at 55–59 years (*P* < 0.001).

**Conclusions:**

This study showed that for women aged 45 years and above who were diagnosed with OEM, the independent risk factors of EAOC were menopausal status, tumor size of 8 cm or greater in diameter, and coexisting endometrial disorders. Therefore, intensive follow-ups or active interventions should be considered for them.

## Background

Endometriosis is a common gynecologic condition with an estimated incidence of 10–15% in women of reproductive age and 25% of postmenopausal age [1–3]. Albeit a benign disease, endometriosis has consistently been associated with an increased risk of developing ovarian cancer [4–6]. Besides the strong association between endometriosis and the increased risk of ovarian cancer, our prior studies found that EAOC represents a distinct clinical entity with more favorable biological behavior, a lower stage distribution and better survival than non-EAOC [7, 8]. Based on this point, researchers have intention to explore if there are any factors which helped to identify EAOC high-risk candidates from general population of patients with endometriosis [9–12]. Many scholars have indicated some risk factors according to their data, but they did not reach a consensus [13, 14]. And these studies had different inclusion criteria of endometriosis which might bias their conclusion, for example, by self-reported or ultrasonographically diagnosed [13–16].

According to prior studies, older age at endometriosis diagnosis (≥45 years) is associated with an increased risk of epithelial ovarian carcinoma among women with endometriosis [14], and the average age of patients with EAOC at diagnosis was 45–50 years [8, 17, 18]. Therefore, we retrospectively studied the medical records of women aged 45 years and above who were diagnosed with OEM by both surgical and pathological examinations.

## Methods

By reviewing the medical records, we retrospectively identified 1038 patients aged 45 years and above who were Surgical-pathologically diagnosed with OEM. All patients received surgery and treatment at the Department of Obstetrics and Gynecology in Peking Union Medical College Hospital from December 1996 through December 2016. Clinical features were studied according to medical records. The study protocol was approved by the Institutional Review Board of the Peking Union Medical College.

In this study, “endometriosis-associated ovarian cancer” was defined as epithelial ovarian cancer coexisting with endometriotic cysts in the same or contralateral ovary confirmed by histology. Based on the criteria listed above, pathologists confirmed that 30 patients were identified as EAOC (group 1) and the remaining 1008 patients as ordinary OEM (group 2). In group 1, 17 patients (56.7%, 17/30) critically met Scott’s criteria for endometriosis associated ovarian cancer [19, 20]. Among the remaining 13 patients (44.3%, 13/30), 7 were found to have ovarian cancer and coexisting endometriosis in the same ovary (no contiguous area between benign and malignant lesions was found however), while the other 6 patients were found to have malignant lesion in the contra-lateral ovary of endometriosis. The revised 2014 FIGO staging system for epithelial ovarian cancer was adopted in this study [21].

Continuous variables were analyzed using an independent-sample *t* test. Categorical variables were analyzed using test or Fisher’s exact test and odds ratio (OR) and 95% confidence interval (CI) were reported. The multivariate analysis about the relationship between the clinicopathological characteristics and the incidence of development of ovarian cancer was assessed by logistic regression models. Variables included in this analysis were those found to be statistically significant in the independent-sample *t* test and Fisher’s exact test (as listed in Table 1). Receiver Operating Characteristic (ROC) curve was constructed to define the optimal cutoff value for stratifying and grouping. SPSS 22.0 was used for analyses. All the *P* values reported were two-tailed and a *P* value of less than 0.05 was regarded as statistically significant.

**Table 1:** Comparison of Variables between EAOC and OEM groups

Variable	EAOC	OEM	P	OR	95% CI
Total	30	1008			
Age					
Mean ± SD(y)	50.8 ± 4.1	48.5 ± 3.9	.002		
Range(y)	45–59	45–80			
<50y [n (%)]	13 (43.3%)	738 (73.2%)	<.001	3.57	1.71–7.46
≥ 50y [n (%)]	17 (56.7%)	270 (26.8)			
Menarche age, y			.987		
Mean ± SD(y)	13.9 ± 1.4	13.9 ± 1.6			
Range(y)	11–17	10–23			
Menopausal status [n (%)]			<.001	5.50	2.54–11.90
YES	11 (36.7)	96 (9.5)			
No	19 (63.3)	912 (90.5)			
Gravidity(times)					
Mean ± SD	2.3 ± 1.2	2.2 ± 1.3	0.671		
Range(y)	0–5	0–7			
0	2 (6.7)	55 (5.5)			
1	3 (10.0)	250 (24.8)			
2	11 (36.7)	313 (31.1)			
≥ 3	11 (36.7)	381 (37.8)			
Unknown	3 (10.0)	9 (0.9)			
Parity (times)			.963		
Mean ± SD	1 ± 0.4	1 ± 0.5			
Range(y)	0–2	0–4			
0	2 (6.7)	98 (9.7)			
1	26 (86.7)	812 (80.6)			
2	2 (6.6)	89 (8.8)			
≥ 3	0 (0)	7 (0.7)			
Unknown	0 (0)	2 (0.2)			
Main symptoms [n (%)]					
Pelvic mass incidentally found	10 (33.3)	342 (33.9)	1.000		
Dysmenorrhea^a^	1 (3.3)	293 (29.1)	.011	0.12	0.02–0.88
Irregular abdominal pain	13 (43.3)	186 (18.5)	.001	3.38	1.61–7.08
Irregular menstruation/ Abnormal vaginal bleeding	5 (16.7)	147 (14.6)	.792		
Others	1 (3.3)	40 (4.0)	.860		
Ca125 before surgery(U/mL) Number[n (%)] 27(90)779(77.3)					
Median, IQR	57.1, 32.7 ~ 168.4	55.9, 24.6 ~ 78.4	.091		
Range	11–1468	0.5–4736			
<35	9 (33.3)	358 (46.0)	.240		
≥ 35	18 (66.7)	421 (54.0)			
Tumor size^b^ (cm)					
Number[n (%)]	29(96.7)	933(92.6)			
Mean ± SD	8.5 ± 4.3	5.4 ± 2.9	.001		
Range	3–20	1–30			
<8 cm	11 (37.9)	760 (81.5)	<.001	7.19	3.34–15.50
≥ 8 cm	18 (62.1)	173 (18.5)			
Coexisting myoma or adenomyosis [n (%)]			1.000		
Yes	21 (70.0)	688 (68.3)			
No	9 (30.0)	320 (31.7)			
Coexisting endomentrium disorders [n (%)]			.003	4.11	1.73–9.73
Number[n (%)]	28 (93.3)	665(66)			
Yes	8 (28.6)	59 (8.9)			
No	20 (71.4)	606 (91.1)			

*Abbreviation: EAOC* endometriosis-associated ovarian cancer, *OEM* ovarian endometriosis, *OR* odds ratio, *CI* confidence interval, *SD* standard deviation, *IQR* InterQuartile Range

^a^ This part was only compared among women in premenopausal status

^b^ This part was only compared among women having a visible tumor in the surgery, *n* = 29 in group 1; *n* = 933 in group 2

## Results

Based on the pathological diagnosis mentioned above, the prevalence of EAOC among women aged over 45 years with OEM was 2.9% (30/1038). Overall, the mean age of the 1038 women was 48.6 ± 3.90 years (range, 45–80 years). The clinical and pathological data were compared between the two groups as shown in Table 1. The greatest diameter of the tumors was measured during surgery, and there were 29 (96.7%, 29/30) patients in group 1 and 933 (92.6%, 933/1008) patients in group 2 who were found to have visible tumor during the surgery. For those with multiple and isolated lesions, the diameter of the largest lesion was considered.

As seen in Table 1, patient’s age, menopausal status, clinical manifestation at the first hospital visit, along with, coexisting endometrial disorders, and tumor size were significantly associated with EAOC. Patients with EAOC were approximately 2 years older than those with OEM. (50.8 vs. 48.5 years, *P* = .002). In addition, we studied the prevalence of EAOC in this series at different onset age: 45–49 years, 1.7% (13/751); 50–54 years, 5.6% (12/215); 55–59 years, 10.0% (5/50). No patient with EAOC was over 59 years.

Besides, 10.3% (107/1038) of patients were in postmenopausal status. Postmenopausal women occupied 36.7% (11/30) in group 1 while 9.5% (96/1008) in group 2(*P* < .001). For women with endometriosis and aged over 45 years, postmenopausal patients were associated with 5.50-fold increased risk of EAOC than premenopausal ones (OR 5.50, 95%CI 2.54–11.90). Moreover, among premenopausal women, EAOC was less likely to be found in patients whose chief complaint was dysmenorrhea (OR 0.12, 95% CI 0.02–0.88, *P* = .011), while EAOC occurred more often in patients with irregular abdominal pain as the chief complaint (OR 3.38, 95% CI 1.61–7.08, *P* = .001).

Furthermore, analysis by ROC curve indicated that 8 cm should be considered as a cutoff value for tumor size when evaluating the risk of EAOC in patients with OEM. As a result, it showed the positive correlation between tumor size of ≥8 cm and the possibility of EAOC (62.1% vs. 18.5%, *P* < .001). Compared to patients with smaller tumor size, those with tumor size of 8 cm and above were found to have 7.19-fold higher risk of developing EAOC (OR 7.19, 95% CI 3.34–15.50).

Meanwhile, the coexisting endometrial disorders were studied among patients who received hysterectomy or diagnostic uterine curettage, along with OEM surgery at the same time[*n* = 28 (93.3%, 28/30 in group 1, and *n* = 665 (66.0%, 665/1008) in group 2]. As seen in Table 2, the coexisting endometrial disorders included endometrial polyp (EP), endometrial hyperplasia (EH), atypical endometrial hyperplasia (AH), atypical polypoid adenomyoma and endometrial cancer (EC)). To be precise, the diagnosis of synchronous ovarian cancer and endometrial cancer should meet the criteria defined by Young and Scully [22]. A significant difference was found in the prevalence of coexisting endometrial disorders between two groups (26.7% vs 5.9%, *P* = 0.03). In this series, OEM patients with coexisting endometrial disorders were found to have 4.11-fold higher risk of developing EAOC, compared to those without coexisting endometrial disorders. (OR 4.11, 95%CI 1.73–9.73). As seen in Table 2, the distribution of all histological types of the coexisting endometrial disorders showed no significant difference between two groups, except for endometrial hyperplasia (EH) except for EH.

**Table 2:** The coexisting endometrial disorders in EAOC and EOM groups

Pathology of coexisting endometrial disorder	EAOC	OEM	P
Total number	8	59	
EP	4^a^(50.0%)	47^c^(79.7%)	0.085
EH	3^b^(37.5%)	2 (3.4%)	0.010
AH	3 (37.5%)	11 (18.6%)	0.349
APA	1(12.5%)	1 (1.7%)	0.226
EC	1(12.5%)	0 (0)	

*Abbreviation: EP* endometrial polyp, *EH* endometrial hyperplasia, *AH* atypical endometrial hyperplasia, *APA* atypical polypoid adenomyoma, *EC* endometrial cancer

^a^ including 1 patients with both EP and EH, and another with both EP and AH

^b^ including 2 patients with both EH and AH

^c^ including 1 patients with both EP and EH, and another with both EP and AH

**Fig. 1:**
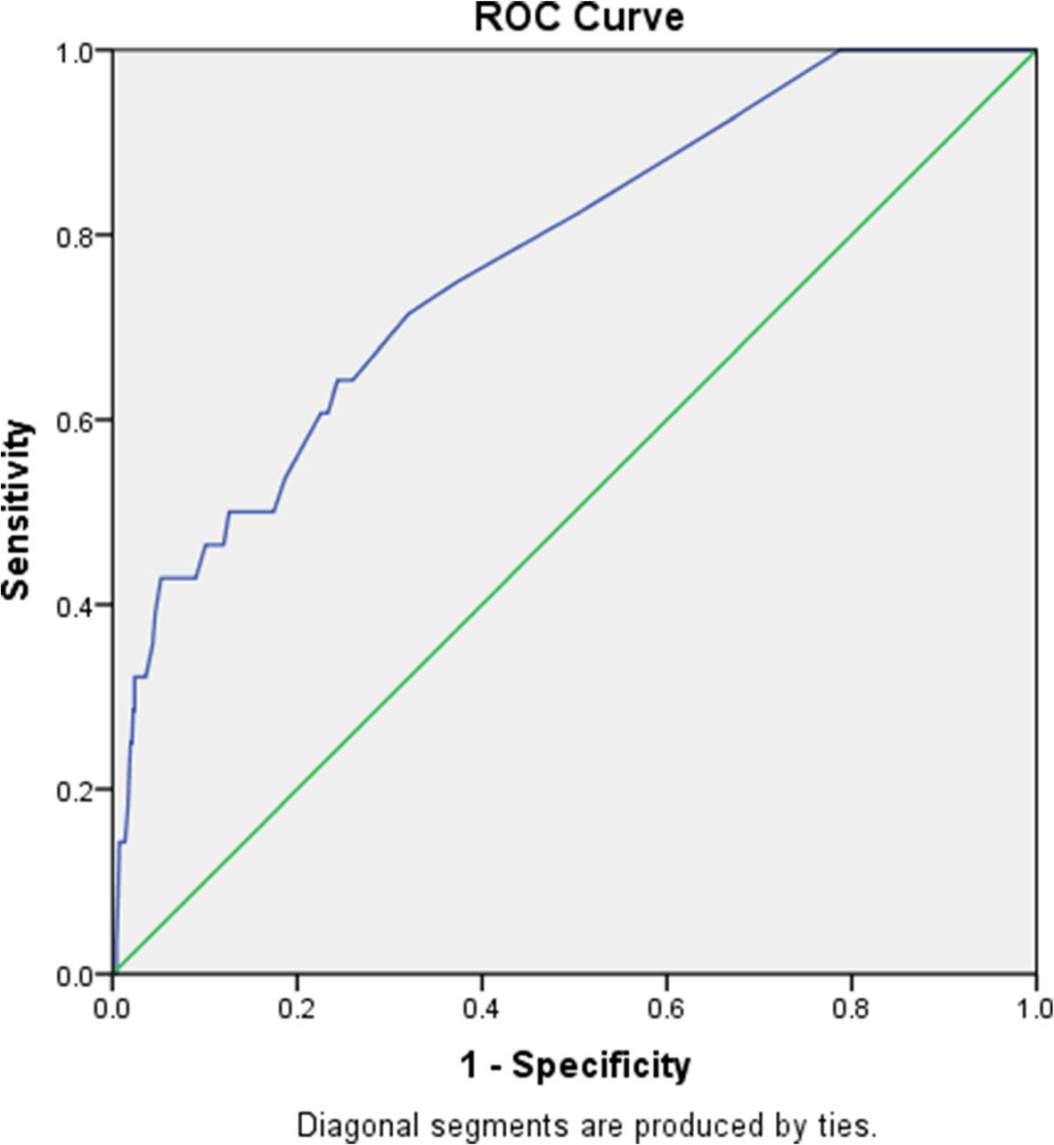
ROC curve to build a predictive model for risk of EAOC among patients with OEM

There were no differences in menarche age, times of gravidity and parity, and coexisting uterine myoma or adenomyosis between the two groups (Table 1). Interestingly, either the mean value of preoperative serum CA125 or the proportion of patients with Ca125 in normal range showed no significant difference between two groups (for group 1 and 2, median (InterQuartile Range, IQR), 57.1(32.7–168.4) vs 55.9(24.6 ~ 78.4)U/mL, *P* = .091; 33.3% vs 46%, *P* = .240). Thereafter, we enrolled all patients obtaining the pathological diagnosis of coexisting endometrial tissue (*n* = 693) into the multivariate analysis, which indicated that menopausal status, tumor size and coexisting with endometrial disorder were found to be the independent predictors of EOAC, as seen in Table 3. Accordingly, we tried to plot a ROC curve to build a predictive model for risk of EAOC in this series (as seen in Fig. 1). The accuracy, sensitivity and specificity of this cutoff value (at postmenopausal status, with the tumor size ≥8 cm and coexisting with endometrial disorders) were respectively 75.2, 64.3 and 75.6% in detecting EAOC among patients with OEM (AUC 0.774, 95%CI, 0.685 ~ 0.863, *P* < .001).

**Table 3:** Multivariate analysis of risk factors of EAOC for OEM patients ≥ 45years^a^

Variable	Category	EAOC		
		OR	95% CI	P
Age	≥50	1.243	0.461–3.348	0.668
	<50			
Postmenopausal status	Yes	3.099	1.050–9.145	0.041*
	No			
Tumor size	≥8 cm	6.566	2.950–14.615	<0.001*
	<8 cm			
Coexisting endometrial disorder	Yes	3.053	1.181–7.889	0.021*
	No			

^a^ The multivariate analysis included all patients who had obtained the pathological diagnosis of coexisting endometrial tissue (*n* = 693)

The distributions of International Federation of Gynecology and Obstetrics (FIGO) stage and histological subtypes of EAOC are presented in Table 4. In this study, 70.0% (21/30) of subjects in EAOC group were diagnosed at FIGO stage I. Clear cell and endometrioid ovarian subtypes occupied 90.0% (27/30) of EAOC.

**Table 4:** Distribution of FIGO Stage (2014) [21] and Histologic Subtypes of EAOC

Characteristics	Number	Percentage
Total	30	
FIGO stage		
I	22	73.3
II	3	10.0
III	4	13.4
IV	1	3.3
Histology		
Clear cell	19	63.3
Endometrioid	7	23.3
HGSC	2	6.7
Mixed	2	6.7
CCC + EAC	1	
LGSC + CCC	1	

*Abbreviation: FIGO* International Federation of Gynecology and Obstetrics, *EAOC* endometriosis-associated ovarian cancer, *HGSC* high-grade serous carcinoma, *CCC* clear cell carcinoma, *EAC* endomtrioid adenocarcinoma, *LGSC* low-grade serous carcinoma

## Discussions

The previous clinical and pathological data suggested EAOC might be a distinct clinical entity, and it was proposed that the pathogenesis of EAOC is distinct from that of non-EAOC [7]. And the recent findings by molecular biological studies have accumulated the evidence of the association between endometriosis and ovarian cancer [9, 14, 23]. Some scholars considered that endometriosis is the precancerous lesion of ovarian clear cell and endometrioid carcinoma. There were few of studies intending to explore the potential risk factors of ovarian cancer for women with endometriosis [14–16]. However, these studies could not provide us with the common consensus due to their limitations and bias. Therefore, in this study, we retrospectively analyzed the data of women aged 45 years and above who were surgically and pathologically diagnosed with OEM. The reason why we intend to focus on the women aged 45 years and above has been mentioned in the section of Background.

In present study, the total prevalence of EAOC in patients with ovarian endometriosis was 2.9%, which was higher than that in previous reports on women at either all ages or over 45 years (0.3–1.7%) [6, 9, 24]. This discrepancy might be owing to the different inclusion criteria either for endometriosis or EAOC within the studies. The diagnosis of all subjects chosen in this study were confirmed by both surgery and histology, unlike some studies which were based on ultrasound finding, patients’ symptoms, even retrospective self-report. On the other hand, EAOC group in this study was defined as the coexistence of endometriosis and the malignant tumor in the same or the contralateral ovary, but not critically alliance to Scott’s criteria, although 56.7% of our patients in EAOC group met the latter standard. The comparatively strict inclusion standard of endometriosis and the probably wider diagnostic criteria of EAOC might lead to a higher prevalence of ovarian cancer related endometriosis. Lee et al. had also demonstrated that the risk of EOC of women with endometriosis varies greatly with different diagnostic criteria, and a bias of selected subjects might significantly influence the risk estimation [13, 14]. Because lacking of precise and non-invasive diagnostic tool, studying the risk of cancer among patients with endometriosis whose diagnose had been surgical-pathologically defined might be more valuable to help us to make clinical strategy at this moment.

In this study, patients with EAOC were older than those with benign endometriosis (50.8 vs. 48.5 years, *P* = .002). The results also showed the significant tendency of climbing-up prevalence of EAOC as patient’s age is increasing, 1.7% at the age of 45–49 years, 5.6% at 50–54 years, and 10.0% at 55–59 years. These findings were in agreement with previous study on patients at all ages [25]. Although this result might be biased in some degree due to the range of patients age (45–60 years), it provided us with the valuable information that more attention should be paid to the elder patients on the issue of malignant change of endometriosis, and it might be necessary to built the age-stratified strategy for screening the patients of higher EAOC risk in future.

We wonder whether the patients of benign OEM group had underwent earlier and more regular check-ups, rather than seeing a doctor only after experiencing discomforts, when compared with ones of EAOC group. However, our data showed no significant difference between two groups in the proportion of patients who were incidentally found the ovarian mass with no symptoms. It seemed more likely that EAOC has some intrinsic characters which make it distinct from ordinary endometriosis. That means there might be certain peculiarities in EAOC patients.

Our data revealed that EAOC patients were more likely to be at post-menopausal status. It has been reported that 35% of patients diagnosed with endometriosis at menopausal status were found to have metaplasia, hyperplasia, atypia changes and endometrioid carcinoma in ovarian lesions. 7% of them developed EAOC, which ratio was lower than the prevalence in our study (11.5%, 11/96) [26, 27]. And our data showed postmenopausal patients were associated with 5.50-fold increased risk of EAOC than premenopausal ones (OR 5.50, 95%CI 2.54–11.90). Some researchers proposed that exogenous hormone intake might induce the malignant change of endometriosis for postmenopausal women. However, in our cohort, no patients with EAOC had received hormone therapy after menopause. We assumed that the endometriotic lesions developed a specific carcinogenic pathway to transform into ovarian cancer. And they were less dependent on estrogen than benign endometriosis. Future studies on the mechanism of malignant changes in postmenopausal endometriosis might help us to better understand this condition. This study also indicated that among premenopausal women, dysmenorrheal might be a protective factor for risk of EAOC (OR 0.12, *P* = .011); whereas for all women aged 45 years and above, EAOC occurred more often in patients with irregular abdominal pain.

Another finding in our study was that EAOC patients had the higher frequency of concurrent endometrial disorders than those with isolated endometriosis (26.7% vs 5.9%, *P* = 0.003). The endometrial disorders mentioned above were analyzed according to the specimen of patients who had received hysterectomy or diagnostic uterine curettage (66.8%, 693/1038). Among the remaining patients (33.2%, 345/1038) whose endometrial specimens were not obtained by hysterectomy or curettage surgery, most of them (87.2%) had no related clinical manifestations, such as irregular menstruation, abnormal vaginal bleeding, or ultrasound findings prompting potential endometrial abnormalities. If we included the patients who had symptoms of endometrial disorders but no histological proof into the combing endometrial disorder category, the risk of EAOC was still found to be higher among those with concurrent endometrial disorders, when compared to those with isolated endometriosis (*P* = 0.01). All in all, our data suggested that more attention should be paid to patients with concurrent endometrial disorders when screening EAOC. Some other studies had also revealed the possible association between EAOC and its uterine counterpart. And accordingly, some scholars preferred to raise the interesting question that EAOC be induced by “bad endometriosis or bad endometrium” [28]. The in-depth molecular biological exploration for EAOC lesion and the courterpart eutopic endometrium would be benefit for us to clarify this issue.

It had been well-documented that women with endometriosis have increasing risk for infertility, and infertility and nulliparity per se are associated with higher risk of developing ovarian cancer [29–32]. However, our results showed OEM patients who were infertile (*P* = .658) or nulliparous (*P* = .761) had the same risk of EAOC as that of fertile or multiparous women. Similarly, Melin et al. reported that women with endometriosis had an increased risk for ovarian cancer, which was not influenced by gravity or parity [16]. However, Wang et al. indicated that the risk of developing ovarian cancer for endometriosis patients was higher in infertile women than fertile women. But the undifferentiated carcinoma and carcinosarcoma were included in their study, and these subtypes were not usually seen as endometriosis-associated ovarian cancer.

Unexpectedly, the pre-operative serum value of Ca125 showed no significant difference for endometriosis patients with or without EAOC in this series. And also no difference was seen between the two groups for the ratio of patients with normal range value. Identically, our prior study showed that patients with EAOC were more likely to present with normal range Ca125 than patients with non-EAOC. Based on these findings, it was proposed that preoperative Ca125 value might be a poor marker for distinguishing EAOC from benign OEM. As such, the malignant possibility should not be underestimated for OEM patients with normal value of preoperative CA125.

Furthermore, our data also showed the positive correlation between tumor size of ≥8 cm and the possibility of EAOC (62.1% vs. 18.5%, *P* < .001), those patients had 7.19-fold higher risk of EAOC compared to those with <8 cm tumors. Our results were accordant with the findings of the previous cohort study, the author reported that the subgroup of patients who were postmenopausal and with a larger size of endometrioma (>8 cm) at the time of diagnosis had an elevated risk of ovarian cancer [9].

Additionally, in our sample, Multivariate analysis showed that menopausal status, tumor size ≥8 cm and coexisting with endometrial disorders were independent predictive factors of EAOC for OEM patients at 45 years or above. Based on our data, the predictive model built by combining the aforementioned three factors showed the accuracy, sensitivity and specificity of 75.2, 64.3 and 75.6% respectively in detecting EAOC among patients with OEM. However, given the limitation of retrospective design and the data based on single institution in this study, the results should be considered with caution.

As Table 4 showed, most of patients with EAOC in this series were in early stage (FIGO I and II possessed 83.3%), which is identical with the results of our previous studies [7, 8]. And the clear cell carcinoma and endometriosis occupied 63.3 and 23.3% respectively in all histological types, which also was accordant with literatures.

As a retrospective study, our results might have some limitations and bias. Further large-scale and prospective studies have being planned by our research team.

## Conclusion

Conclusively, the findings in this study indicated the potential risk factors of EAOC for women with ovarian endometriosis (OEM) aged 45 years and above in China. In this series, women at postmenopausal status, with larger size of endometrioma (≥8 cm), and having coexisting endometrial disorders had higher risk of EAOC. On the contrary, preoperative Ca125 value might be a poor marker in either early detecting the malignant change of OEM, or distinguishing EAOC from benign OEM. Accordingly, more active interventions and intensive follow-ups should be considered seriously for this subgroup population.

## Abbreviations

Ca125: Cancer antigen 125
EAOC: Endometriosis-associated ovarian cancer
OEM: Ovarian endometriosis
ROC: Receiver Operating Characteristic
FIGO: International Federation of Gynecology and Obstetrics
